# Real-world evidence of the impact of polypharmacy on mortality and recovery after hip fracture in elderly patients

**DOI:** 10.1038/s41598-026-52300-5

**Published:** 2026-05-08

**Authors:** Elisa García-Tercero, Alejandro Valcuende-Rosique, Ana Valcuende-Rosique, Daniela Andrea Villalon Rubio, Ana Navalon Bono, Cristina Cunha-Pérez, José Viña Ribes, Francisco José Tarazona-Santabalbina

**Affiliations:** 1 Geriatric Medicine Department, Hospital Universitario de La Ribera, Carretera de Corbera Km. 1, 46600 Alzira (Valencia), Spain; 2 Department of Pharmacy, Hospital Universitario de La Ribera, Carretera de Corbera Km. 1, 46600 Alzira (Valencia), Spain; 3 Medical School, Universidad Católica de Valencia San Vicente Mártir, 46001 Valencia, Spain; 4 School of Doctorate, Universidad Católica de Valencia San Vicente Mártir, 46001 Valencia, Spain; 5 Departament of Physiology, Universitat de Valencia, 46010 Valencia, Spain; 6 Centro de Investigación Biomédica en Red Fragilidad y Envejecimiento Saludable (CIBERFES), 28029 Madrid, Spain

**Keywords:** Polypharmacy, Postoperative mortality, Hip fractures, Medication management, Geriatric assessment, Diseases, Health care, Medical research, Risk factors

## Abstract

Polypharmacy is a common problem in older adults with hip fractures and may negatively affect postoperative outcomes. The aim of this study was to evaluate the association between polypharmacy, including severe polypharmacy, and clinical outcomes in older adults admitted to hospital with hip fracture. A real-life observational study was conducted at a tertiary care hospital in Spain, including patients aged ≥ 70 years who underwent hip fracture surgery between January 1, 2017, and December 31, 2018. Data were extracted from electronic medical records, including demographic details, comorbidities, and medication use. Polypharmacy was defined as the use of five or more medications, and severe polypharmacy as the use of ten or more medications. Mortality rates were analyzed at 30 days, 6 months, 1 year, 2 years, and 5 years post-surgery using Kaplan–Meier survival curves and Cox regression analysis. Among 644 patients included (mean age 84.5 years, 70.5% women), 63.8% had polypharmacy and 19.1% had severe polypharmacy. Compared with patients without polypharmacy, those with polypharmacy, regardless of severity, showed higher mortality at 30 days (8.4% and 10.3% vs 3.9%), 6 months (21.3% and 21.4% vs 10.8%), 1 year (26.6% and 33.3% vs 11.6%), 2 years (38.8% and 46.0% vs 14.2%), and 5 years (68.5% and 76.2% vs 26.3%) (all *p* ≤ 0.05). Crude hazard ratios for 5-year mortality were 3.65 (95% CI 2.73–4.88) for patients taking 5–9 drugs and 4.51 (95% CI 3.26–6.24) for those taking ≥ 10 drugs; after full adjustment, these remained 3.12 and 3.46, respectively. Patients with polypharmacy also had more red blood cell transfusions, major complications, and worse functional recovery. Polypharmacy was associated with worse postoperative morbidity, poorer functional recovery, and higher mortality in older adults with hip fracture. These findings suggest that medication burden may serve as a marker of clinical vulnerability rather than an isolated causal factor. Prospective interventional studies are needed to determine whether medication optimization improves outcomes.

## Introduction

Population ageing is increasing the burden of chronic disease, multimorbidity, frailty, and surgery in older adults, with important consequences for postoperative morbidity and mortality. Hip fracture is one of the most frequent and clinically relevant conditions in this population, and its incidence rises sharply with age^1–3^. Beyond the acute surgical event, hip fracture is associated with substantial loss of function, institutionalization, and increased mortality, making it a major challenge for healthcare systems.

Older adults admitted with hip fracture frequently present with frailty, functional and cognitive impairment, malnutrition, and multiple comorbidities, all of which are associated with worse hospital outcomes, poorer recovery, and reduced survival^4–10^. In this context, comprehensive geriatric assessment and orthogeriatric care models are important for optimizing perioperative management, reducing complications, and improving functional recovery. Medication review is a key component of this approach, given the high burden of chronic treatment and the complexity of prescribing in this population.

Polypharmacy and potentially inappropriate prescribing are highly prevalent in older adults and have been associated with adverse drug events, falls, readmissions, delayed recovery, and mortality^11–15^. In patients with hip fracture, these risks may be amplified by frailty, acute illness, and multimorbidity. Polypharmacy may therefore represent not only a marker of treatment burden, but also a clinically relevant indicator of vulnerability and poorer prognosis after surgery. Severe polypharmacy, commonly defined as the use of 10 or more medications, may identify a subgroup with even greater clinical complexity and risk, although its prognostic value in comparison with polypharmacy overall remains insufficiently characterized^12,13^.

Furthermore, the impact of polypharmacy in older adults with hip fracture is likely to extend beyond mortality alone. Previous studies suggest that multiple medication use may also be associated with impaired functional recovery, prolonged rehabilitation, and greater healthcare utilization after surgery^14^. However, the available evidence remains limited and heterogeneous, particularly with regard to long-term outcomes.

Despite increasing recognition of polypharmacy as a marker of poor prognosis in older adults, few studies have specifically examined its impact in patients admitted with hip fracture. In particular, data remain scarce on the comparison between patients without polypharmacy and those with polypharmacy, including those with severe polypharmacy, across short-, medium-, and long-term outcomes^16,17^. Moreover, limited evidence is available on the specific medication classes that may contribute most to adverse outcomes after surgery.

The present study aimed to evaluate the association between polypharmacy status and clinical outcomes in older adults undergoing surgery for hip fracture, comparing patients without polypharmacy, with polypharmacy, and with severe polypharmacy. Specifically, we examined the relationship between these medication-burden categories and postoperative complications, functional recovery, and short-, medium-, and long-term mortality, as well as the contribution of selected drug classes to 5-year mortality risk.

## Methods

### Study design

This real-life observational study was conducted at a tertiary care hospital in Spain, using a hospital-based database. It included patients aged 70 years or older who underwent surgical treatment for hip fracture between January 1, 2017, and December 31, 2018. Upon admission, each patient was managed by a multidisciplinary orthogeriatric team consisting of a traumatologist, a geriatrician, and a specialized nursing team. In addition, clinical pharmacists reviewed the patients’ chronic home medication regimens to identify potential medication-related problems, including therapeutic duplications, potentially inappropriate medications, clinically relevant drug–drug interactions, and opportunities for treatment optimization. The geriatrician and traumatologist assessed patients within the first 24 h of admission and daily thereafter. After surgery, rehabilitation was initiated within 48 h. The traumatologist determined the surgical approach, whereas the geriatrician performed a comprehensive geriatric assessment addressing medical, functional, cognitive, nutritional, and social domains. Comorbidities were evaluated to inform perioperative management. For patients with cognitive impairment, additional information was obtained from caregivers. Discharge decisions were made jointly by the traumatologist, geriatrician, and rehabilitation specialist. When indicated, rehabilitation was continued after discharge at referral rehabilitation facilities. This care model was designed to provide early, comprehensive, and coordinated management, with particular emphasis on prompt geriatric assessment, individualized surgical decision-making, and early rehabilitation to promote recovery of mobility after surgery^18^.

### Study participants

All patients aged 70 years or older who underwent surgical hospitalization for hip fracture were eligible for inclusion in this study. Inclusion criteria encompassed patients aged 70 years or older who were admitted specifically due to hip fracture. Exclusion criteria included polytrauma, pathological fracture, and an estimated life expectancy of less than six months from any cause. Patients were classified according to the number of medications prescribed at hospital discharge into three groups: no polypharmacy, polypharmacy (5–9 medications), and severe polypharmacy (≥ 10 medications)^19,20^.

### Outcomes

The study outcomes included short-term (1 month) and medium-term (6 months) functional recovery, as well as short-term, medium-term, and long-term mortality at 1, 2, and 5 years. In addition, adverse events, complications during hospitalization, and length of hospital stay were assessed according to polypharmacy status.

### Study variables

Two co-investigators, blinded to the study design and objectives, were responsible for data collection. The variables assessed included sociodemographic characteristics (age and sex), surgical delay, length of hospital stay, comorbidity measured by the Charlson Comorbidity Index (CCI) and age-adjusted CCI^21^, frailty assessed using the Clinical Frailty Scale (CFS), potentially inappropriate prescribing according to START/STOPP criteria, nutritional status assessed with the Controlling Nutritional Status (CONUT) scale^22^, geriatric syndromes such as delirium, red blood cell transfusions, and medical and surgical complications during hospitalization. Mortality outcomes were also evaluated.

### Data collection

Data were obtained from the electronic clinical records of hospitalized patients, including inpatient records, outpatient prescribing records, the hospital’s electronic prescribing system, and hospital discharge summaries. Follow-up information was collected from outpatient records, emergency care episodes, and post-discharge hospital admissions. Survival information, including deaths occurring within the hospital’s catchment area, was obtained from updated medical records. Polypharmacy status was determined according to the number of medications prescribed at hospital discharge. Medication count was established by reviewing the discharge treatment regimen documented in the electronic medical record. All data were subsequently entered into an anonymized database for analysis.

### Statistical analysis

The statistical analysis was conducted using IBM SPSS Statistics® version 23. A power analysis estimated a 16.9% difference in 30-day post-discharge mortality among patients with hip fracture, corresponding to a statistical power of 99.6%^23^. Categorical variables were summarized as frequencies and percentages, while quantitative variables were described using measures of central tendency (mean or median) and dispersion (standard deviation or interquartile range).

Normality of quantitative variables was assessed using the Kolmogorov–Smirnov test. Variables with a normal distribution were summarized as means and standard deviations, whereas non-normally distributed variables were described as medians and interquartile ranges.

Categorical variables were compared using the Pearson chi-square test, and quantitative variables with a normal distribution were compared using analysis of variance (ANOVA). Five-year survival was evaluated using the non-parametric Kaplan–Meier method, and differences according to polypharmacy status were assessed with the log-rank test. Multivariable Cox proportional hazards regression models were then fitted to examine the association between polypharmacy and mortality. Crude hazard ratios (HRs) and progressively adjusted models were estimated, including sex, age, CCI Index, relevant medical history, prescription of specific pharmacological groups, and START/STOPP criteria. Before model fitting, potential collinearity among covariates related to multimorbidity, functional status, and nutritional status was assessed using correlation matrix analysis and variance inflation factors. No evidence of problematic collinearity was identified, and variables with clinical relevance were retained in the final models. In addition, the number of outcome events relative to the number of covariates was considered adequate to support model stability. Statistical significance was defined as *p* < 0.05 for all tests.

### Ethical considerations

This study was conducted in accordance with the ethical principles of the Declaration of Helsinki and complied with Spanish Organic Law 3/2018 on the Protection of Personal Data and Guarantee of Digital Rights. The study protocol was approved by the institutional Ethics and Clinical Research Committee (approval code HULR23122022).

Given the retrospective nature of the study and the use of anonymized data extracted from electronic medical records, the requirement for informed consent was waived by the institutional Ethics and Clinical Research Committee, in accordance with current Spanish legislation. No direct patient contact occurred, no personally identifiable information was collected, and no interventions were performed on the patients. All data were handled confidentially and stored in a secure, anonymized database exclusively for scientific research purposes.

## Results

The study enrolled 644 older adults admitted to the hospital with hip fracture, of whom 70.5% (n = 454) were women, with a mean age of 84.5 years (SD 6.1). Polypharmacy was present in 63.8% (n = 411) of patients, whereas severe polypharmacy was observed in 19.1% (n = 123).

Baseline characteristics of the patients stratified by polypharmacy status (no polypharmacy, polypharmacy, and severe polypharmacy) are presented in Table 1a. Clinical variables collected during hospitalization and follow-up are shown in Table 1b. The study identified a significant association between polypharmacy status and increased mortality across all analysed intervals: 30 days (10.3% severe polypharmacy vs 8.4% polypharmacy vs 3.9% no polypharmacy; *p* = 0.043), 6 months (21.4% vs 21.3% vs 10.8%; *p* = 0.003), 1 year (33.3% vs 26.6% vs 11.6%; *p* < 0.001), 2 years (46.0% vs 38.8% vs 14.2%; *p* < 0.001) and 5 years (76.2% vs 68.5% vs 26.3%; *p* < 0.001). As shown in Table 2, the crude mortality data in the three categories is higher in severe polypharmacy and polypharmacy than in no polypharmacy at all times measured. Table 3 shows the standardized results for multiple variables.

**Table 1:** **a** Diagnostic characteristics between patients with and without polypharmacy. *CCI* score Charlson index score. *CONUT* Lale. *Hx of IHD* history of ischemic heart disease. *Hx of HF* history of heart failure. *Hx of COPD* history of chronic obstructive pulmonary disease. *Hx of CVA* history of cerebrovascular accident. *Hx of RF* history of renal failure. *m*  mean; *SD* standard deviation. **b** Bivariate Analysis of variables analysed during hospital stay. *ICU* intensive care unit, *UTI* urinary tract infection. *Hb* Hemoglobin. *m* mean; *SD* standard deviation.

Variable	No polypharmacy N = 232	Polypharmacy N = 286	Severe polypharmacy N = 126	*p* value
(a)				
Age (years), m (SD)	83.2 (6.3)	84.3 (6.0)	85.5 (6.0)	< 0.001
Male sex, n (%)	57 (24.6%)	82 (28.7%)	51 (40.5%)	0.006
CCI score, m (SD)	1.7 (1.8)	2.9 (2.4)	4.0 ± (2.4)	< 0.001
CONUT at hospital admission score in range of moderate or severe risk of malnutrition, n (%)	152 (72.7%)	182 (78.1%)	86 (82.7%)	0.122
Number of START criteria, m (SD)	0.5 (0.7)	1.4 (0.9)	2.1 (0.9)	< 0.001
Number of STOPP criteria, m (SD)	0.3 (0.5)	0.6 (0.7)	1.1 (0.9)	< 0.001
Hx of IHD, n (%)	10 (4.3%)	26 (9.1%)	25 (19.8%)	< 0.001
Hx of HF, n (%)	6 (2.6%)	29 (10.1%)	11 (8.7%)	0.003
Hx of COPD, n (%)	22 (9.5%)	30 (10.5%)	29 (23.0%)	< 0.001
Hx of CVA, n (%)	3 (1.3%)	17 (5.9%)	3 (2.4%)	0.013
Hx of Dementia, n (%)	28 (12.1%)	43 (15.0%)	15 (11.9%)	0.533
Hx of RF, n (%)	12 (5.2%)	27 (9.4%)	19 (15.1%)	0.007
Hx of Diabetes, n (%)	48 (20.7%)	78 (27.3%)	68 (54.0%)	< 0.001
(b)				
ICU, n (%)	2 (0.9%)	4 (1.4%)	4 (3.2%)	0.230
In-hospital mortality rate, n (%)	5 (2.2%)	7 (2.4%)	7 (5.6%)	0.101
CONUT at hospital discharge, n (%)	119 (60.7%)	143 (67.5%)	65 (71.4%)	0.153
Complications, n (%)	92 (39.7%)	159 (55.6%)	74 (58.7%)	< 0.001
Major complications, n (%)	87 (37.5%)	152 (53.1%)	73 (57.9%)	< 0.001
Delirium, n (%)	19 (8.2%)	18 (6.3%)	12 (9.5%)	0.479
AE Cardiac, n (%)	12 (5.2%)	19 (6.6%)	13 (10.3%)	0.180
AE Anemia, n (%)	19 (8.2%)	28 (9.8%)	14 (11.1%)	0.644
AE UTI, n (%)	6 (2.6%)	7 (2.4%)	2 (1.6%)	0.823
AE Digestive Issues, n (%)	0 (0.0%)	3 (1.0%)	2 (1.6%)	0.205
AE Respiratory, n (%)	7 (3.0%)	15 (5.2%)	8 (6.3%)	0.295
Surgey Delay in hours, m (SD)	40.1 (28.8)	44.3 (26.9)	45.5 (25.4)	0.110
Surgery Delay of 48 Hours, n (%)	167 (72.0%)	184 (64.3%)	77 (61.1%)	0.068
Surgery Delay of 72 Hours, n (%)	205 (88.4%)	248 (86.7%)	106 (84.1%)	0.527
Red blood cells Trasfusions, n (%)	119 (51.3%)	178 (62.2%)	78 (61.9%)	0.028
Initial Hb, m (SD)	12.8 (1.7)	12.1 (1.7)	12.3 (1.5)	< 0.001
Final Hb, m (SD)	10.3 (1.1)	10.4 (1.2)	10.3 (1.2)	0.526
Initial Albumin (g/dL)	4.0 (0.4)	3.9 (0.4)	3.9 (0.4)	0.056
Final Albumin (g/dL)	3.8 (0.5)	3.7 (0.5)	3.6 (0.5)	0.026
Initial Glomerular Filtration (mil/min), m (SD)	70.1 (22.1)	63.5(25.1)	58.0 (23.8)	< 0.001
Time from hospital discharge to first readmission in days., m(SD)	657.6 (614.0)	474.7 (579.9)	465.3 (556.7)	0.015
Final Glomerular Filtration (mil/min), m (SD)	87.8 (35.1)	80.2 (35.8)	73.6 (36.0)	< 0.001
Readmissions after Hospital Discharge, n (%)	139 (59.9%)	195(68.2%)	94(74.6%)	0.004
Readmissions after Hospital Discharge, m (SD)	1.6 (2.0)	1.8 (2.0)	2.7 (3,0)	< 0.001
Walking at thirty days, n (%)	181 (84.6%)	151 (59.4%)	63 (58.3%)	< 0.001
Walking at six months, n (%)	185 (90.2%)	169 (73.8%)	68 (70.1%)	< 0.001

**Table 2:** Crude data of mortality. No polypharmacy, polypharmacy and severe polypharmacy.

	No polypharmacy N = 232	Polypharmacy N = 286	Severe polypharmacy N = 126	*p* value
30-day mortality, n (%)	9 (3.9%)	24 (8.4%)	13 (10.3%)	0.043
180-day mortality, n (%)	25 (10.8%)	61 (21.3%)	27 (21.4%)	0.003
365-day mortality, n (%)	27 (11.6%)	76 (26.6%)	42 (33.3%)	< 0.001
2-year mortality, n (%)	33 (14.2%)	111 (38.8%)	58 (46.0%)	< 0.001
5-year mortality, n (%)	61 (26.3%)	196 (68.5%)	96 (76.2%)	< 0.001

**Table 3:** Cox regression. * = Adjusted Rate of Polypharmacy and Severe Polypharmacy by sex, age and Charlson Comorbidity Index; **† = **Adjusted Rate of Polypharmacy and Severe Polypharmacy by sex, Charlson Comorbidity Index, History of Ischemic Heart Disease; History of Heart Failure; History of Chronic Obstructive Pulmonary Disease; History of Cerebrovascular Accident; History of Renal Failure; History of Diabetes; **‡ = **Adjusted Rate of Polypharmacy and Severe Polypharmacy by Start criteria, STOPP criteria; benzodiazepines prescription; neuroleptic prescription; hypotensives prescription; dementia treatment prescription; opioids prescription; anti-diabetcs drugs prescription. **¥ = **Adjusted Rate of Polypharmacy and Severe Polypharmacy by all previous variables used.

Variable	Hazard ratio	95%CI	*p* value
*Crude rate of polypharmacy and severe polypharmacy*			
Polypharmacy 5–9 drugs	3.65	2.73–4.88	< 0.001
Polypharmacy ≥ 10 drugs	4.51	3.26–6.24	< 0.001

Variable *	Hazard ratio*	95%CI	*p* value
Adjusted rate of polypharmacy and severe polypharmacy*			
Polypharmacy 5–9 drugs	2.90	2.15–3.91	< 0.001
Polypharmacy ≥ 10 drugs	3.33	2.37–4.68	< 0.001

Variable	Hazard ratio†	95%CI	*p* value
*Adjusted rate of polypharmacy and severe polypharmacy†*			
Polypharmacy 5–9 drugs	2.82	2.08–3.82	< 0.001
Polypharmacy ≥ 10 drugs	3.13	2.20–4.45	< 0.001

Variable	Hazard ratio‡	95%CI	*p* value
*Adjusted rate of polypharmacy and severe polypharmacy ‡*			
Polypharmacy 5–9 drugs	2.44	1.72–3.46	< 0.001
Polypharmacy ≥ 10 drugs	2.55	1.61–4.05	< 0.001

Variable	Hazard ratio¥	95%CI	*p* value
*Adjusted rate of polypharmacy and severe polypharmacy ¥*			
Polypharmacy 5–9 drugs	3.12	2.24–4.34	< 0.001
Polypharmacy ≥ 10 drugs	3.46	2.24–5.34	< 0.001

**Fig. 1:**
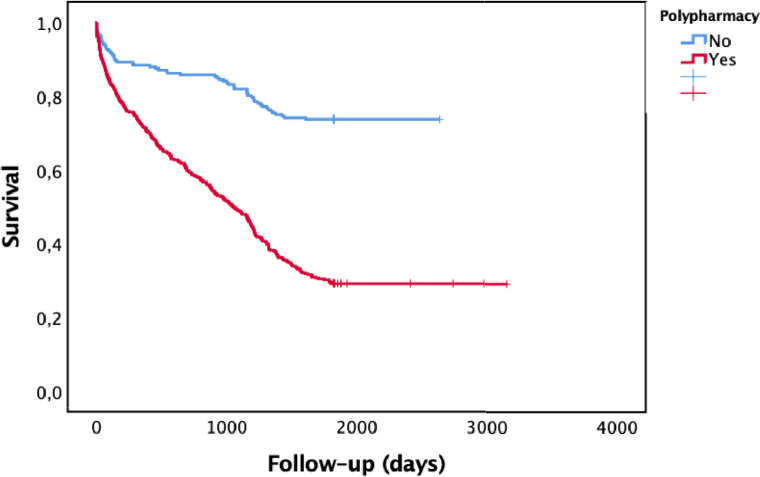
Survival curves of patients with polypharmacy and without polypharmacy. Kaplan–Meier method.

**Fig. 2:**
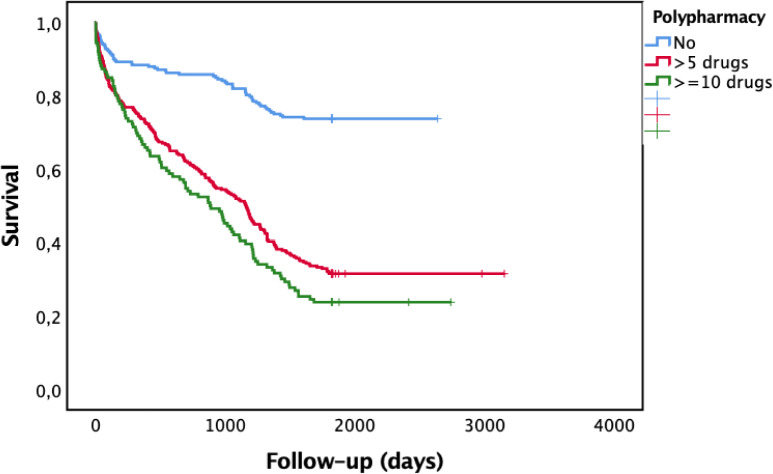
Survival curves of patients without polypharmacy, with polypharmacy, and with severe polypharmacy. Kaplan–Meier method.

Regarding the medication classes analyzed, adjusted associations with 5-year mortality were observed for benzodiazepines/hypnotics, antihypertensives, neuroleptics, anti-dementia drugs, opioids, and antidiabetics. The corresponding adjusted hazard ratios, derived from Cox regression models adjusted for sex, age, and CCI, are shown in Table 4. Kaplan–Meier survival analysis also demonstrated an association between polypharmacy status and survival (Figs. 1 and 2). START/STOPP criteria were applied to identify potentially inappropriate prescribing across patients without polypharmacy, with polypharmacy, and with severe polypharmacy. A higher number of inappropriate medications was observed in patients with polypharmacy than in those without polypharmacy (*p* < 0.001). In exploratory analyses adjusted for sex, age, and CCI, the use of specific drug classes was associated with increased 5-year mortality, including antihypertensives (HR 1.83, 95% CI 1.45–2.32), benzodiazepines/hypnotics (HR 1.69, 95% CI 1.37–2.08), antidiabetics (HR 1.83, 95% CI 1.46–2.29), opioids (HR 1.46, 95% CI 1.11–1.90), neuroleptics (HR 1.91, 95% CI 1.46–2.51), and anti-dementia drugs (HR 1.92, 95% CI 1.36–2.69).

**Table 4:** Association between selected drug classes and 5-year mortality by Cox regression, adjusted for sex, age, and Charlson Comorbidity Index.

Drugs	Means of the covariates	Hazard ratio	Hazard ratio 95% CI	*p* value
Benzodiazepines	0.41	1.69	1.37–2.08	< 0.001
Hypotensive agents	0.64	1.83	1.45–2.32	< 0.001
Neuroleptics	0.13	1.91	1.46–2.51	< 0.001
Antidementia drugs	0.07	1.92	1.36–2.69	< 0.001
Opiates	0.15	1.46	1.11–1.90	0.006
Antidiabetics	0.24	1.83	1.46–2.29	< 0.001

These findings further support an association between polypharmacy and higher 5-year mortality in older adults with hip fracture. Compared with patients without polypharmacy, 5-year mortality risk was significantly higher in those with polypharmacy, regardless of severity (*p* < 0.001). The crude HR was 3.65 (95% CI 2.73–4.88) for patients taking 5–9 medications and 4.51 (95% CI 3.26–6.24) for those taking ≥ 10 medications. After full adjustment, the corresponding HRs were 3.12 (95% CI 2.24–4.34) and 3.46 (95% CI 2.24–5.34), respectively.

Consistent with these findings, multivariable Cox regression analysis showed that patients without polypharmacy had a longer 5-year mean survival than those with polypharmacy or severe polypharmacy (66.21 [SD 1.99] months vs 47.60 [SD 2.41] and 36.95 [SD 2.94] months, respectively). As shown in Fig. 2, no statistically significant differences were observed between polypharmacy and severe polypharmacy at the individual mortality time points of 30 days, 6 months, 1 year, 2 years, and 5 years.

Functional recovery was poorer in patients with severe polypharmacy and polypharmacy than in those without polypharmacy, as reflected by lower rates of walking recovery at 1 and 6 months after hospitalization (Table 1b). Readmission burden also differed according to polypharmacy status. Patients with severe polypharmacy and polypharmacy had a shorter time to first readmission and a higher number of readmissions after discharge than those without polypharmacy. However, no significant differences were observed between severe polypharmacy and polypharmacy for these outcomes.

Additionally, patients with polypharmacy, whether severe or non-severe, had higher rates of red blood cell transfusions, total complications, and major complications than those without polypharmacy. Red blood cell transfusion rates were 61.9% in severe polypharmacy, 62.2% in polypharmacy, and 51.3% in no polypharmacy (*p* = 0.028). Total complication rates were 58.7%, 55.6%, and 39.7%, respectively (*p* < 0.001), and major complication rates were 57.9%, 53.1%, and 37.5%, respectively (*p* < 0.001). CCI scores were also higher in patients with severe polypharmacy and polypharmacy than in those without polypharmacy, with mean (SD) values of 4.0 (2.4), 2.9 (2.4), and 1.7 (1.8), respectively (*p* < 0.001). By contrast, polypharmacy status was not associated with longer hospital stay, delayed hip surgery, or a higher incidence of delirium during hospitalization.

## Discussion

Polypharmacy was associated with a clinically meaningful worsening of prognosis in older adults admitted with hip fracture. Patients taking five or more chronic medications had higher short-, medium-, and long-term mortality, poorer recovery of ambulation, and more in-hospital complications than those without polypharmacy. Although severe polypharmacy was associated with the highest absolute risk, the main prognostic contrast was between any polypharmacy and no polypharmacy, whereas the gradient between 5–9 and ≥ 10 medications was more modest. Taken together, these findings support the interpretation of polypharmacy as a clinically relevant marker of vulnerability and poorer postoperative trajectory in this population. Our results are consistent with previous studies linking polypharmacy to adverse outcomes in older adults, including mortality, complications, and impaired recovery^11,14,19,24^.

The association between polypharmacy and adverse outcomes in older adults has been widely reported^25–28^, extending beyond immediate postoperative risk to encompass longer-term health consequences^29^. In our cohort, polypharmacy was highly prevalent, affecting 63.8% of patients, and was associated with a greater burden of comorbidity and in-hospital complications. Previous studies have also linked polypharmacy to both an increased risk of hip fracture and poorer postoperative outcomes, including higher mortality^16,30^. In this setting, polypharmacy may therefore be regarded not only as a measure of treatment burden, but also as a marker of clinical complexity that warrants careful assessment in older adults undergoing surgery for hip fracture.

The coexistence of polypharmacy, multimorbidity, and frailty makes the management of these patients particularly challenging^15,31,32^. In our study, any degree of polypharmacy was associated with higher rates of red blood cell transfusions, total complications, and major complications, as well as poorer functional recovery and greater readmission burden. By contrast, no significant associations were observed with length of hospital stay, surgical delay, or delirium during hospitalization. This pattern suggests that medication burden may be more closely related to the severity and complexity of the postoperative course than to hospitalization duration itself.

Our findings also underscore the importance of comprehensive geriatric assessment in the management of older adults with hip fracture. Comprehensive geriatric assessment facilitates the early identification of frailty, comorbidities, and specific patient needs^6,9^. By integrating medical, functional, psychological, and social domains, it may support individualized care planning and more appropriate medication review in this complex population. Effective coordination between geriatricians, pharmacists, and other specialists is essential to address polypharmacy and its potential complications^15,33^. Although the specific contribution of this orthogeriatric approach to the outcomes observed in the present study cannot be determined, such care models are likely to be particularly relevant in patients with high treatment burden.

Polypharmacy was consistently associated with higher mortality at 30 days, 6 months, 1 year, 2 years, and 5 years after hip fracture surgery, and this association persisted after multivariable adjustment. However, severe polypharmacy in our cohort also likely reflected a greater burden of multimorbidity, frailty, and clinical complexity. Patients with severe polypharmacy were older and had poorer baseline clinical profiles, supporting the interpretation of polypharmacy as both a prognostic marker and an indicator of global vulnerability. Accordingly, although medication burden may plausibly contribute directly to adverse outcomes through drug–drug interactions, treatment complexity, and medication-related harm, the retrospective design does not allow the independent effect of polypharmacy to be fully separated from the underlying burden of disease.

In our cohort, the use of antihypertensives, benzodiazepines/hypnotics, antidiabetics, opioids, neuroleptics, and anti-dementia drugs remained associated with higher 5-year mortality after adjustment for sex, age, and CCI index. These findings should be interpreted cautiously, as they are susceptible to confounding by indication and likely reflect both treatment burden and underlying clinical complexity^34^. Nevertheless, these drug classes may help identify patients at particularly high risk and may therefore be useful in guiding more careful medication review. Whether deprescribing or medication optimization alone can improve mortality or functional recovery in this setting remains uncertain and requires prospective interventional studies^11,35^. In this context, a multidisciplinary approach involving geriatricians, primary care physicians, pharmacists, and other specialists may help improve prescribing appropriateness and treatment individualization. Tools such as STOPP/START criteria and the Beers Criteria may also support the identification of potentially inappropriate medications^36^. Notably, severe polypharmacy was also associated with poorer functional recovery, an outcome that has been less extensively studied to date.

Several limitations should be considered when interpreting these findings. First, the retrospective design precludes causal inference between polypharmacy and mortality. The observed associations may partly reflect baseline differences in clinical status, as patients with polypharmacy had a greater burden of comorbidity, frailty, and older age. Although multivariable adjustment was performed, residual confounding cannot be excluded, and it was not possible to fully disentangle the effect of severe polypharmacy from the underlying burden of multimorbidity and clinical vulnerability. Second, polypharmacy status was defined at a single time point, according to the number of medications prescribed at hospital discharge, and changes in medication burden during follow-up were not captured. Therefore, the potential influence of deprescribing or other longitudinal modifications in treatment on long-term mortality could not be evaluated. Third, although loss to follow-up was relatively low (7%), it may still have introduced bias. Potential adverse effects related to home medications were not recorded, and although medication exposure was assessed using electronic prescribing records and patient/caregiver interview, actual adherence to home treatment could not be objectively confirmed. In addition, while medication review by clinical pharmacists formed part of routine care, the nature and extent of these interventions were not systematically documented, and their potential influence on study outcomes could not be assessed. Finally, the number of modified START/STOPP criteria and the number of medications discontinued at discharge were not collected.

Despite these limitations, our findings support the clinical relevance of polypharmacy as a marker of vulnerability in older adults with hip fracture. Identifying patients with high medication burden may help support more individualized multidisciplinary care and more careful review of potentially modifiable prescribing-related risks. Future research should focus on prospective and interventional approaches to clarify whether medication optimization can improve clinical outcomes in this population.

## Conclusions

Polypharmacy was associated with worse postoperative morbidity, poorer functional recovery, and higher mortality among older adults with hip fracture. These findings suggest that medication burden may serve as a marker of clinical vulnerability in this population. Identifying patients with polypharmacy may help support comprehensive medication review and individualized multidisciplinary care. However, given the observational design of the study, these results should be interpreted as evidence of association and prospective interventional studies are needed to determine whether medication optimization improves clinical outcomes.

## Data Availability

The datasets used and analyzed during the current study are available from the corresponding author on reasonable request.
